# Comparison of Motion Grading in 1,000 Patients by First- and Second-Generation HR-pQCT: A Propensity Score Matched Cohort Study

**DOI:** 10.1007/s00223-023-01143-7

**Published:** 2023-10-25

**Authors:** Mikolaj Bartosik, Alexander Simon, André Strahl, Ralf Oheim, Michael Amling, Felix N. Schmidt

**Affiliations:** 1 Department of Osteology and Biomechanics, University Medical Center Hamburg-Eppendorf, Hamburg, Germany; 2 Division of Orthopaedics, Department of Trauma and Orthopaedic Surgery, University Medical Center Hamburg-Eppendorf, Hamburg, Germany

**Keywords:** High-resolution peripheral quantitative computed tomography, Motion grading, Motion artifacts, Microarchitecture, Muscle performance

## Abstract

*In-vivo* bone microstructure measured by high-resolution peripheral quantitative computed tomography (HR-pQCT) is gaining importance in research and clinical practice. Second-generation HR-pQCT (XCT2) shows improved image quality and shorter measurement duration compared to the first generation (XCT1). Predicting and understanding the occurrence of motion artifacts is crucial for clinical practice. We retrospectively analyzed data from HR-pQCT measurements at the distal radius and tibia of 1,000 patients (aged 20 to 89) evenly distributed between both generations of HR-pQCT. Motion artifacts were graded between 1 (no motion) and 5 (severe motion), with grades greater 3 considered unusable. Additionally, baseline characteristics and patients’ muscle performance and balance were measured. Various group comparisons between the two generations of HR-pQCT and regression analyses between patient characteristics and motion grading were performed. The study groups of XCT1 and XCT2 did not differ by age (XCT1: 64.9 *vs.* XCT2: 63.8 years, *p* = 0.136), sex (both 74.5% females, *p* > 0.999), or BMI (both 24.2 kg/m^2^, *p* = 0.911) after propensity score matching. XCT2 scans exhibited significantly lower motion grading in both extremities compared to XCT1 (Radius: *p* < 0.001; Tibia: *p* = 0.002). In XCT2 motion-corrupted scans were more than halved at the radius (XCT1: 35.3% *vs.* XCT2: 15.5%, *p* < 0.001), and at the tibia the frequency of best image quality scans was increased (XCT1: 50.2% *vs.* XCT2: 63.7%, *p* < 0.001). The strongest independent predictor for motion-corrupted images is the occurrence of high motion grading at the other scanning site during the same consultation. The association between high motion grading in one scan and a corresponding high motion grading in another scan within the same session suggests a non-resting patient. Additionally, aged, female, and patients with smaller stature tend towards higher motion grading, requiring special attention to a correct extremity fixation.

**Supplementary Information:**

The online version contains supplementary material available at 10.1007/s00223-023-01143-7.

## Introduction

**Fig. 1:**
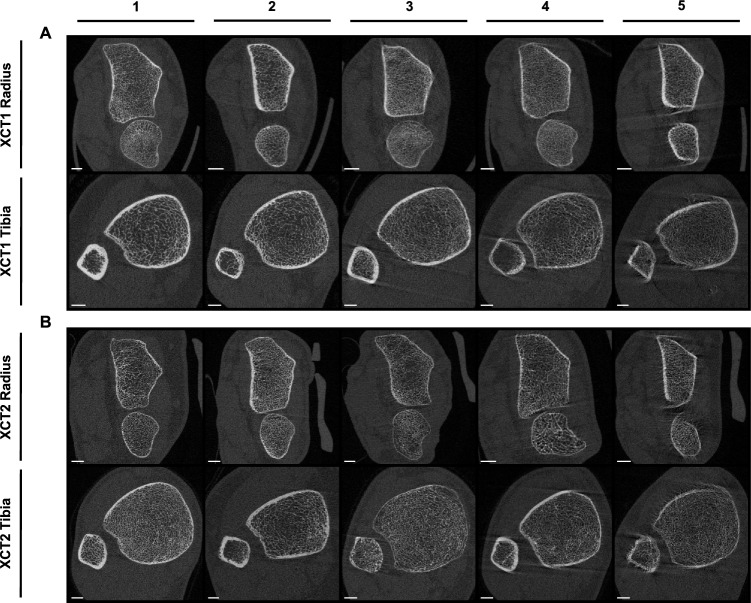
HR-pQCT image grading scale for motion artifact. Motion artifacts are classified into five grades (1 ≙ none, 2 ≙ minor, 3 ≙ moderate, 4 ≙ severe, and 5 ≙ extreme) for first-generation HR-pQCT (**A**) and second-generation HR-pQCT (**B**). White bars in the lower left corner of each image correspond to 5 mm length

High-resolution peripheral quantitative computed tomography (HR-pQCT) enables to assess the bone microstructure and mineral content non-invasively. Therefore, it can provide additional critical information in fracture prediction [1, 2] and facilitates a deeper understanding of metabolic processes of the bone, compared to two-dimensional bone densitometry (DXA) [3, 4]. However, due to the very high-resolution *in-vivo* (XCT1: 142.2 μm spatial resolution at 82 µm isometric voxel size; XCT2: 95.2 μm spatial resolution at 60.7 μm voxel size [5]) and thereby long scanning and integration time (XCT1: 2.8 min; XCT2: 2.0 min [6]), HR-pQCT measurements are in general more sensitive to patient movements compared to standard clinical computed tomography. Motion artifacts during the measurement are usually caused by coughing or talking, tremors, twitching, or muscle contractions, which decrease the validity of the bone parameters. The extent to which motion artifacts distort bone parameters particularly falsifies structural parameters of the trabecular bone [7, 8]. Motion artifacts are usually classified into five grades from 1 to 5 (Fig. 1), whereby the higher the grade, the more severe motion artifacts are present [6]. The grading scales are identical for first-generation (XCT1) and second-generation HR-pQCT (XCT2). While bone density parameters appear to be relatively resilient to motion [7, 8], bone microstructure parameters should not be interpreted for measurements with a motion grading higher than 3. Particularly, trabecular structural parameters are susceptible to motion artifacts [7]. Therefore, measurements with a motion grading of 4 and 5 are inadequate for full evaluation and should be repeated [6, 9]. Despite low irradiation dose, a reduction of repeated measurements is always targeted to reduce the irradiation burden to the patient [6, 10].

As shown in previous studies, motion artifacts which lead to repeated measurements in XCT1 occur in up to 30% of patients at the radius and are less present at the tibia [7]. An improvement with respect to motion grading can be expected in XCT2 due to a 28.6% reduction of the scanning time (XCT1: 2.8 min vs. XCT2: 2.0 min) [6]. Furthermore, an adapted forearm cast could provide more stability due to a different fixation. However, the question remains as to what extent both structural alterations and changes in scanning time of the XCT2 can lead to minimizing the number of motion-corrupted scans.

Considering the close connection between musculature and motion, it would be of great interest to determine whether motion artifacts can be predicted based on a muscle performance and balance assessment prior to scanning. In the future, this could potentially aid in identifying patients who need to be scanned with special care and precautions.

## Materials and Methods

### Study Design

This study was conducted as a retrospective cross-sectional study in accordance with local guidelines and the Declaration of Helsinki. 1,000 patients visiting our outpatient clinic (*Department of Osteology and Biomechanics*) were examined in clinical routine, with 500 patients in each generation of HR-pQCT. Patients’ demographics were recorded, and examinations including DXA and HR-pQCT were performed. In addition, muscle and balance tests were performed to detect sarcopenia and imbalance. For each patient, all measurements were performed on the same day and motion grading was evaluated by three independent experts in each case (MB, AS, FNS).

### Dual-Energy X-Ray Absorptiometry (DXA)

The areal bone mineral density (aBMD, with T- and Z-score) was measured by DXA (Lunar iDXA, GE Healthcare, Madison, WI, USA). The entire lumbar spine (L1-L4) and both sides (left/right) of the hip (femoral neck and total hip) were examined. For further analysis, the T-score (with corresponding absolute BMD and Z-score) of L1-L4 and the lowest T-score of both hip examinations (with corresponding absolute BMD and Z-score) were used. For DXA quality control, calibration scans were acquired daily using a specialized phantom according to the manufacturer's recommendations. This included accuracy tests including least significant change calculations following the guidelines of the International Society for Clinical Densitometry (ISCD) [11].

### High-Resolution Peripheral Quantitative Computed Tomography (HR-pQCT) and Motion Grading

Patients were scanned with either first- or second-generation HR-pQCT (XtremeCT and XtremeCT II, Scanco Medical AG, Brüttisellen, Switzerland) at the non-dominant distal radius and the contralateral distal tibia using the protocol of standard *in-vivo* scanning for each HR-pQCT (XCT1: 59.4 kVp, 900 μA, 100 ms integration time, 82.0 μm voxel size; XCT2: 68.0 kVp, 1,470 μA, 43 ms integration time, 60.7 μm voxel size). The scan region extends over 110 slices for XCT1 and 168 slices for XCT2, representing a total scan region of 9.02 mm and 10.20 mm in length, respectively. The scan region starts at a fixed offset distance from the insertion point of the end plate of the distal radius or the tibial plafond and extends proximally from this. The fixed offset distance is 9.5 mm at the radius and 22.5 mm at the tibia for XCT1 and 9.0 mm and 22.0 mm for XCT2, respectively. Measurements were conducted in accordance with Whittier et al. [6]. Patients were examined by a group of trained technicians to minimize operator bias. The extremities of the patients were fixed in a device-specific cast provided by the manufacturer using hook-and-loop tape. Patients were asked to remain calm, not to talk, and to visually fix a point in the direction of view.

Volumetric bone mineral density (vBMD) was expressed as total BMD (Tt.BMD, mg HA/cm^3^), cortical BMD (Ct.BMD, mg HA/cm^3^), and trabecular BMD (Tb.BMD, mg HA/cm^3^). Microarchitecture parameters followed the standardized nomenclature of the IOF-ASBMR-ECTS working group [6] and included bone volume-to-total volume ratio (BV/TV), trabecular number (Tb.N, mm^−1^), trabecular thickness (Tb.Th, mm), trabecular separation (Tb.Sp, mm), cortical thickness (Ct.Th, mm), and cortical porosity (Ct.Po, %). Geometric values included total bone area (Tt.Ar, mm^2^), trabecular bone area (Tb.Ar, mm^2^), cortical bone area (Ct.Ar, mm^2^), and cortical perimeter (Ct.Pm, mm). HR-pQCT results were compared with device-, age-, and sex-specific reference values [12, 13].

The scans for each patient (radius and tibia) were evaluated using the manufacturer’s image quality grading scale for motion artifacts (1 ≙ none, 2 ≙ minor, 3 ≙ moderate, 4 ≙ severe, and 5 ≙ extreme) by three skilled examiners (Fig. 1) [6–8]. Grade 1 shows no visible motion artifacts, while grades 2 to 3 show slight to moderate horizontal streaks but intact cortex continuity. At grade 4 and above, also cortex continuity is at least partially disrupted and trabeculae are smeared. According to the manufacturer’s recommendations, measurements with a motion grading of 4 and 5 are inadequate for evaluation [6, 9]. The discrepancies in motion grading between the three examiners were never greater than one, and if there was a discrepancy, they consulted together and decided on one. Interrater reliability was evaluated by intraclass correlation coefficient (ICC = 0.95, *p* < 0.001) and indicates excellent reliability [14].

### Muscle Performance and Balance

Muscle performance tests included grip strength and chair raising test (CRT). Maximum grip strength was measured using a hand-held dynamometer (Leonardo Mechanograph® GF, Novotec Medical, Pforzheim, Germany) while the patients were seated with their arms resting on their thighs. Three measurements were taken for each arm (left/right), and the highest value was used for further analysis. CRT was performed using Leonardo Mechanograph® (Leonardo Mechanograph® GRFP STD, Novotec Medical, Pforzheim, Germany). Patients were seated on a bench and told to stand up and sit down as quickly as possible for five cycles. Both the maximum force and time per repetition were recorded by the force plate. Balance was assessed by Romberg posturography, also using the Leonardo Mechanograph® GRFP. Patients stood on the force platform with their feet together, arms out in front of them at shoulder height, and were instructed to stand still for ten seconds with their eyes open, testing the balance under visual control. Next, they were asked to repeat the test with their eyes closed in order to test balance ability without visual control. The center of pressure movement was recorded over ten seconds by the ground reaction force platform for both conditions (eyes open and eyes closed) and the corresponding path length (mm) was calculated according to Simon et al. [15]. The presence of sarcopenia was defined using thresholds for low muscle performance based on grip strength and CRT time per repetition, as recommended by the EWGSOP2 consensus [16].

### Statistical Analysis

For statistical analysis, SPSS Statistics 29.0 (IBM, Armonk, NY, USA) and GraphPad Prism 9.5 (GraphPad Software, San Diego, CA, USA) were used. Results are expressed as mean ± standard deviation (SD) and with mean percentage of the median of reference values for HR-pQCT parameters. To evaluate normal distribution of the data, the Shapiro–Wilk test was used. For testing differences between two subgroups, the unpaired two-tailed *t* test was used for normally distributed data and the Mann–Whitney *U* test was used for non-parametric data. When testing for differences between three groups, one-way analysis of variance (ANOVA) with Holm–Šídák test was used for normally distributed data and Kruskal–Wallis *H* test with Dunn’s test for non-parametric data. Differences in the distribution in subgroups were tested by chi-square test. Effect sizes were reported as *r* or *φ* (> 0.1 ≙ small, > 0.3 ≙ medium, > 0.5 ≙ large effect size) [17]. A multiple linear regression model (enter method) was applied to evaluate the predictive value of the independent variables sex, age, and height on radial and tibial motion gradings (dependent variables).

To allow comparability between the two generations of HR-pQCT, we applied propensity score matching for sex, age, BMI, and DXA T-score. As a result, the group size was reduced from 500 to 400 per device. For the further investigation of the device-dependent associations, we included the entire study cohort.

## Results

### Characterization of the Study Cohort

The characteristics of the entire study cohort can be found in Supplementary Table 1. The propensity score matched study cohort with their demographic, densitometric, and mechanographic characteristics is presented in Table 1. After propensity score matching there were almost no significant differences in age, weight, height, BMI, and DXA values between the XCT1 and XCT2 cohorts, despite deviations in spinal T-score and CRT time per repetition but with small effect size (Spinal T-score: −1.6 in XCT1 *vs.* −1.4 in XCT2, *p* = 0.008, *r* = 0.09; CRT time per repetition: 1.94 s in XCT1 *vs.* 2.10 s in XCT2, *p* = 0.003, *r* = 0.11). DXA measurements revealed that most of the patients were within the range of osteoporosis (48.5% in XCT1 and 43.5% in XCT2) and osteopenia (42.3% in XCT1 and 47.5% in XCT2). In addition, we detected sarcopenia in 17.0% and 18.8% of the included patients in XCT1 and XCT2, respectively. Bone microstructure characterization with overall values below the range for age- and sex-specific reference values were found in patients with adequate motion grading (Grades 1 to 3) in the propensity score matched cohort (Supplementary Table 2).

**Table 1:** Overview of the propensity score matched study cohort

Parameter	XCT 1 (*n* = 400)				XCT 2 (*n* = 400)				*p*	*r*|*φ*
	Mean	SD	Min	Max	Mean	SD	Min	Max		
Demographics										
Female (%)	74.5				74.5				> 0.999	0.00
Age (years)	64.9	12.7	20	89	63.8	13.0	21	87	0.136	0.05
Weight (kg)	68.2	15.0	38.2	146.0	68.6	15.2	41.1	155.3	0.755	0.01
Height (m)	1.68	0.09	1.46	1.95	1.68	0.09	1.38	1.98	0.471	0.03
BMI (kg/m^2^)	24.2	4.6	15.4	50.5	24.2	4.6	15.7	54.4	0.911	0.00
DXA										
Spinal T-score	−1.6	1.6	−4.9	4.8	−1.4	1.6	−4.3	4.3	**0.008**	0.09
Spinal Z-score	−0.4	1.6	−3.9	6.4	−0.2	1.6	−3.8	6.4	0.143	0.05
Femoral T-score	−2.1	0.9	−4.1	3.3	−2.0	0.9	−4.8	2.3	0.157	0.05
Femoral Z-score	−0.8	1.0	−3.3	5.1	−0.8	0.9	−4.6	3.0	0.716	0.01
Lowest T-score	−2.3	1.0	−4.9	3.3	−2.3	0.9	−4.8	0.9	0.193	0.05
Lowest Z-score	−1.1	1.1	−3.9	5.1	−1.1	1.0	−4.6	3.0	0.976	0.00
Normal BMD	37 of 400 (9.3%)				36 of 400 (9.0%)				0.902	0.00
Osteopenia (< −1.0)	169 of 400 (42.3%)				190 of 400 (47.5%)				0.135	0.05
Osteoporosis (≤ −2.5)	194 of 400 (48.5%)				174 of 400 (43.5%)				0.156	0.05
Mechanography										
Grip strength (kg)	26.3	9.3	7.6	63.5	26.0	8.9	8.0	61.0	0.529	0.02
CRT maximum force (kN)	0.90	0.20	0.44	1.93	0.89	0.20	0.47	1.97	0.230	0.04
CRT time per repetition (s)	1.94	0.81	0.87	8.30	2.10	0.95	0.82	9.42	**0.003**	0.11
Romberg path length EO (mm)	141.4	57.2	51.7	420.2	139.2	58.5	56.4	408.9	0.313	0.04
Romberg path length EC (mm)	221.6	112.0	68.1	981.9	224.3	113.3	72.0	853.7	0.877	0.01
Normal muscle performance	332 of 400 (83.0%)				325 of 400 (81.3%)				0.518	0.02
Sarcopenia	68 of 400 (17.0%)				75 of 400 (18.8%)					

Normal bone mineral density (BMD), osteopenia, and osteoporosis were categorized based on the T-score. Sarcopenia was classified according to the sarcopenia EWGSOP2 consensus scores [16]

*SD* standard deviation, *BMI* body mass index, *DXA* dual-energy X-ray absorptiometry, *CRT* chair-rising test, *EO* eyes open, *EC* eyes closed

Numbers in bold indicate statistical significance (*p* < 0.05) and effect sizes were reported as *r* or *φ*

### Comparison of Motion Grading in and Between Both Generations of HR-pQCT

**Fig. 2:**
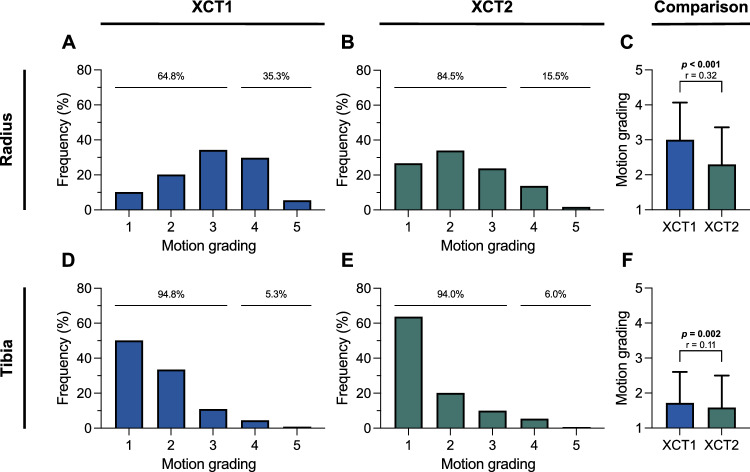
Comparison of motion grading distribution for both generations of HR-pQCT in the propensity score matched cohort. Frequency distribution of motion grading at the distal radius (**A**, **B**) and tibia (**D**, **E**). Comparison of motion grading between the first- and second-generation HR-pQCT at the same site (**C**, **F**). Significant differences in the group comparisons are indicated by exact *p* values with corresponding effect size *r*

At the distal radius, 35.3% of the scans by XCT1 and 15.5% by XCT2 were graded with 4 to 5 and were therefore inadequate for clinical evaluation (*p* < 0.001, *r* = 0.23) (Fig. 2A, B). When comparing motion grading at the distal radius of both device generations, a significantly lower mean motion grading score with medium effect size was found for XCT2 (*p* < 0.001, *r* = 0.32) (Fig. 2C). At the distal tibia, 5.3% of the scans by XCT1 and 6.0% by XCT2 were inadequate for clinical evaluation (*p* = 0.645, *r* = 0.02) (Fig. 2D, E). When comparing mean motion grading at the distal tibia of both device generations a significantly lower motion grading score with small effect size was found for XCT2 (*p* = 0.002, *r* = 0.11) (Fig. 2F). In this context, XCT2 shows more grade 1 scans with 63.8% than XCT1 with 50.3% at the tibial site (*p* < 0.001, *r* = 0.14). Furthermore, mean motion gradings were lower at the tibia compared to the radius in both generations of the HR-pQCT (XCT1: 1.7 at the tibia *vs.* 3.0 at the radius, *p* < 0.001, *r* = 0.54; XCT2: 1.6 at the tibia *vs.* 2.3 at the radius, *p* < 0.001, r = 0.34).

### Association of Demographic, Muscle Performance, and Balance Parameters With Motion Grading

**Fig. 3:**
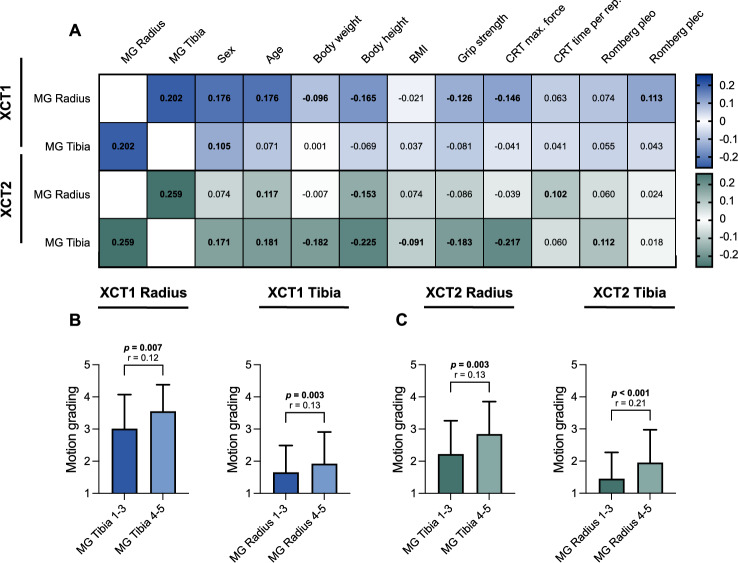
Analyses of motion grading associations in the respective HR-pQCT. Heatmap with correlations of motion grading with the motion grading of the other extremity and demographic, muscle performance, and balance parameters (**A**). Comparison of motion grading in subgroups based on the clinical cut-off value for motion grading (1–3 ≙ adequate, 4–5 ≙ repetition recommended) at the other extremity (**B**, **C**). *MG* Motion grading; *CRT* Chair rising test; *max.* Maximum; *rep*. Repetition, *pleo* Path length eyes open; *plec* Path length eyes closed. Numbers in bold indicate statistical significance (*p* < 0.05). Significant differences in the group comparisons are indicated by exact *p* values with corresponding effect size *r*

For XCT1, significant correlations were found between motion grading at the radius with motion grading at the tibia, sex, age, weight, height, grip strength, and CRT maximum force (Fig. 3A). At the tibia in XCT1, we could show significant correlations between motion grading at the tibia with motion grading at the radius and sex.

For XCT2, significant correlations were found between motion grading at the radius with motion grading at the tibia, age, height, and CRT time per repetition (Fig. 3A). At the tibia in XCT2, significant correlations between motion grading at the tibia with motion grading at the radius and sex, age, weight, height, BMI, grip strength, CRT maximum force, and Romberg path length eyes open were found. While significant correlations between motion grading with the motion grading of the other extremity, female sex, age, CRT time per repetition, and Romberg posturography were positive, significant correlations with weight, height, BMI, grip strength, and CRT maximum force were negative in both device generations. Highest correlations in both device generations were found between the motion gradings of the radius and tibia (XCT1: *r* = 0.202, *p* < 0.001; XCT2: *r* = 0.259, *p* < 0.001).

Patients were classified into two subgroups based on the clinical motion grading cut-off value (Grades ≤ 3 *vs.* Grades ≥ 4) for each extremity, respectively. Significantly higher motion gradings at the other extremity were found in patients in the high motion grading group for radius and tibia in XCT1 (Radius: *p* = 0.007, *r* = 0.12; Tibia: *p* = 0.003, *r* = 0.13) (Fig. 3B) and XCT2 (Radius: *p* = 0.003, *r* = 0.13; Tibia: *p* < 0.001, *r* = 0.21) (Fig. 3C).

### Subgroup-Specific Differences in Motion Grading

**Fig. 4:**
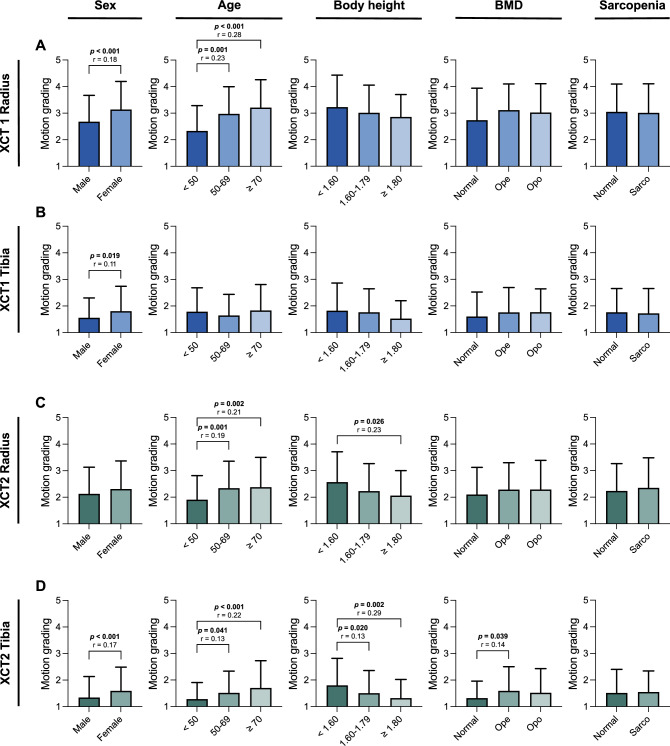
Motion grading in clinical subgroups in both generations of HR-pQCT. Comparison of clinical subgroups regarding motion grading in first- and second-generation HR-pQCT at the distal radius (**A**, **C**) and tibia (**B**, **D**). Significant differences in the group comparisons are indicated by exact *p*-values with corresponding effect size *r*

We subsequently divided our patients into groups for age, sex, body height, bone mineral density, and sarcopenia. For this purpose, we chose the following group distributions: sex according to male and female, age according to < 50 years old, 50–69 years old, and ≥ 70 years old, height according to < 1.60 m, 1.60 m to 1.79 m, and ≥ 1.80 m, bone mineral density according to T-score classified into normal BMD (≥ −1.0), osteopenia (< −1.0 to −2.4), and osteoporosis (≤ −2.5), and muscle performance classified into normal muscle performance and sarcopenia using thresholds based on grip strength and CRT time per repetition, as recommended by the EWGSOP2 consensus [16] (Fig. 4).

At the radius in XCT1, significant differences could be found when comparing sex and age (all *p* ≤ 0.001, *r* = 0.18 to 0.28) with a higher mean motion grading in the female group (Fig. 4A). There were no significant differences present in the groups of height, BMD, and sarcopenia. At the tibia, in XCT1 a significant alteration only in motion grading between the sexes (*p* = 0.019, *r* = 0.11) (Fig. 4B) was detected with a higher mean motion grading in women.

In XCT2 at the radius, significant differences could be shown between the groups in age and height (all *p* < 0.05, *r* = 0.19 to 0.23), but not in sex, BMD, and sarcopenia (Fig. 4C). At the tibia, in XCT2 significant differences were measured in motion grading between the groups in sex, age, height, and BMD (all *p* < 0.05, *r* = 0.13 to 0.29) (Fig. 4D).

Taken together, women and older patients exhibit higher mean motion grading and no differences in motion grading could be detected between the groups with normal muscle performance compared to sarcopenia. In XCT2, additionally, shorter patients tend to exhibit significantly higher motion artifacts. Also, patients with lower bone mineral density tend to show significantly higher motion gradings in the tibial XCT2 scans only.

### Identifying Independent Predictors of Motion Grading

We applied a multiple linear regression model to identify independent predictors of motion grading at the distal radius and tibia for both device generations (Table 2). Therefore, we included the parameters which showed the strongest correlations in our previous analyses. Among these belong sex, age, height, and the motion grading of the other extremity. In total, all multiple linear regression models were significant (*p* < 0.001) and the adjusted R^2^ ranged from 0.039 to 0.105. The motion grading of one extremity proved to be an independent predictor of the motion grading of the other extremity in all cases. Age could also be identified as an independent predictor of the motion grading, but only for the radius in XCT1 and the tibia in XCT2. Moreover, body height could be shown to independently predict the motion grading of the tibia in XCT2, while sex was not an independent predictor of motion grading in any of the sites or device generations.

**Table 2:** Results of multiple linear regression models analyzing independent factors associated with motion grading at the distal radius and tibia in HR-pQCT on both device generations

Parameter	XCT1			XCT2		
	*B*	*ß*	*p*	*B*	*ß*	*p*
Motion grading radius						
Constant	2.165		0.078	3.303		0.005
Sex	0.258	0.100	0.061	−0.050	−0.022	0.693
Age (years)	0.014	0.167	** < 0.001**	0.006	0.085	0.055
Height (m)	−0.382	−0.031	0.561	−1.062	−0.096	0.089
Motion grading tibia	0.205	0.175	** < 0.001**	0.286	0.238	** < 0.001**
	*R*^2^ = 0.092			*R*^2^ = 0.089		
	*R*^2^ adjusted = 0.085			*R*^2^ adjusted = 0.082		
	*F* (4, 495) = 12.612, ***p* < 0.001**			*F* (4, 495) = 12.081, ***p* < 0.001**		
Motion grading tibia						
Constant	1.513		0.160	2.331		0.015
Sex	0.146	0.066	0.224	0.058	0.030	0.579
Age (years)	0.000	0.004	0.930	0.007	0.131	**0.003**
Height (m)	−0.223	−0.021	0.697	−1.021	−0.111	**0.046**
Motion grading radius	0.157	0.184	** < 0.001**	0.193	0.232	** < 0.001**
	*R*^2^ = 0.046			*R*^2^ = 0.112		
	*R*^2^ adjusted = 0.039			*R*^2^ adjusted = 0.105		
	*F* (4, 495) = 6.024, ***p* < 0.001**			*F* (4, 495) = 15.684, ***p* < 0.001**		

*B* and *β* represent unstandardized and standardized regression coefficients, respectively. Next to individual coefficients for each independent variable, overall model characteristics and coefficients are presented for each parameter

Numbers in bold indicate statistical significance (*p* < 0.05)

## Discussion

HR-pQCT is an emerging clinical method to determine fracture risk as well as bone structure and density alterations in bone diseases [18–23] and bone-affecting diseases [24–26]. The modality of bone-structure measurement by HR-pQCT can be well used for non-invasive determination of structural and densitometric bone parameters [27] and has been validated multiple times [5, 28–31]. Yet, motion artifacts are a strong limitation for the evaluation of patients’ bone parameters [6–8]. The aim of this study was to address a comparison of the two generations of HR-pQCT devices with respect to motion artifact occurrence and possible differences between the generations. Changes in the construction (adapted limb fixation for the forearm) and scanning time (28% reduction in XCT2 compared to XCT1) may change the frequency of motion artifacts. Moreover, this study aimed to investigate possible risk factors for motion artifacts by means of clinical and diagnostic patient characteristics.

When comparing motion artifact frequencies and severity in the study cohort of 800 propensity score matched patients, it is evident that XCT2 could certainly improve image quality compared with XCT1. This was particularly the case for the radius, where the number of motion-corrupted images was more than 50% lower on average in XCT2 than in XCT1. Furthermore, a significantly higher number of images with highest image quality was observed in the tibia in XCT2 compared to XCT1. As noted in other studies, grade 4 and 5 motion artifacts occur more frequently with radius measurements than with tibia measurements [7], which was also found for both devices in the present study. Considering the propensity score matched groups for XCT1 and XCT2, it can be assumed that the reduction of motion artifacts is caused by constructional changes. An important aspect may be the reduced scanning time, resulting in 28% less time for potential occurrence of image-corrupting motion artifacts. Minimizing the amount of time the patient has the opportunity to move and cause motion artifacts thus has the greatest potential to reduce artifacts. Furthermore, the adjusted patient fixation for the radius may lead to reduced movements during the scan, which explains the higher number of non-motion-corrupted images.

To examine which patient characteristics were associated with particularly frequent motion artifacts, possible associations of demographic, muscle performance, and balance parameters with motion grading were investigated. This approach can help to ensure special attention is paid to fixation of patients’ extremities who are especially prone to movement during scanning. Significant but low correlations with motion grading were found for both, muscle performance and balance parameters in XCT1 and XCT2. Consistently, strongest motion grading correlations in XCT1 and XCT2 were found with the motion grading of the other limb. This strengthens the assumption that certain patient characteristics lead to increased motion artifacts. In addition to demographics, muscle performance, and balance, this effect may also be caused by the compliance of patients. These patients may tend to bridge an uncomfortable long period of silence during the measurement period by means of conversation. Koudenburg et al. have shown that people can feel uncomfortable even with a pause of four seconds if the flow of conversation seems interrupted [32]. Feelings of distress may cause higher muscle activity in the lumbar region, which could lead to more motion artifacts [33]. Notably, patients experiencing distress due to anxiety did not show significantly more motion in cone beam computed tomography (CBCT) compared to patients without anxiety [34]. Yet, it should be noted that the image resolution in CBCT is significantly lower and therefore less prone to motion artifacts than HR-pQCT (400 µm in CBCT *vs.* 82.0 µm in XCT1 and 60.7 µm in XCT2, respectively). This draws attention to the importance of correct verbalization of instruction for the patients during measurement and the operator to be placed best out of the field of view of the patient during measurement. However, this effect can also be caused by systemic effects and diseases patients may suffer from, such as generalized tremor, Parkinson's disease, or others.

We demonstrated that age was positively correlated with motion grading. Aging is also associated with a loss of muscle mass, up to sarcopenia which points to a possible interaction of muscular conditions and the occurrence of motion artifacts by age [35–37]. Moreover, the prevalence of essential tremor [38, 39] is increasing with age, possibly inducing motion artifacts.

Although muscle volume is directly accessible imaging wise [40], direct muscle performance gives a better clinical image of the muscular capacity of the patient. As a surrogate for muscular performance [16], deteriorations in grip strength and CRT maximum force were found to increase motion artifacts. Therefore, a lack of muscular performance may induce motions through coarse or compensatory movement during the scanning when muscle function is poor and trunk stabilization is insufficient. Nevertheless, we did not find significant differences between patients with normal muscle function and patients with sarcopenia in terms of motion grading.

Furthermore, we could show that female patients exhibit higher motion gradings. However, in the multiple linear regression analysis, it was revealed that sex had no independent predictive value for motion grading of both extremities in both device generations. Because women are on average shorter in height than men [41, 42], collinearities could explain the correlation between female sex and motion grading. Significant negative correlations between motion grading and body height were shown in XCT2 and partially in XCT1 (radius only) as well as higher mean motion grading in shorter patient subgroups. Although we were able to demonstrate the independent predictive value of body height only for the motion grading at the tibia in XCT2, an explanation for this could be the constructions of the device and the greater relative distance of the thorax to the arm/leg fixation mold for tall patients. Therefore, short patients may tend to cause increased movement with movement of the torso.

The fact that the XCT1 exhibits more significant correlations of motion grading at the radius with clinical parameters than the XCT2 may be related to increased susceptibility of the XCT1 to patient movements due to alterations in construction, fixation, and scan duration. However, the XCT2 did exhibit a higher number of significant correlations in the tibia compared to the XCT1, which may be caused by the XCT2 being more specific to the dominating effect of the present confounding clinical condition.

We are aware that this study has limitations and strengths. Based on the retrospective cross-sectional design of the study, associations could be examined, but it is not feasible to derive conclusions regarding causality. Further longitudinal studies are needed to confirm the potential predictors and perhaps identify additional ones. To our knowledge, there is no study to date that has compared and investigated both device generations regarding motion artifacts and also included several clinical parameters such as bone mineral density and muscle performance assessment. In addition, we performed a propensity score matching to minimize the influence of the acquired clinical parameters with respect to motion grading and to ensure optimal comparability between the two device generations.

In conclusion, XCT2 exhibits remarkably reduced frequencies of motion-corrupted images most likely caused by a faster scanning time and changes in extremity fixation. This reduces needed irradiation exposure to patients and increases reliability of bone measures by HR-pQCT. According to the presented results, the strongest parameter for motion grading prediction is a present motion artifact in a recorded image of the same patient. Furthermore, aged, female, and shorter patients tend towards higher motion gradings, drawing particular attention to a correct fixation of the extremity to achieve valid image quality for further processing.

### Supplementary Information

Below is the link to the electronic supplementary material.

Supplementary file1 (DOCX 43 kb)
