# Supplementary material for: Comparison of Motion Grading in 1,000 Patients by First- and Second-Generation HR-pQCT: A Propensity Score Matched Cohort Study
Source: Calcif Tissue Int. 2023 Oct 25;113(6):597–608. doi: 10.1007/s00223-023-01143-7 (PMC10673987; doi:10.1007/s00223-023-01143-7)
Supplement: Supplementary file 1 — Supplementary file1 (DOCX 43 kb) [file 223_2023_1143_MOESM1_ESM.docx]

**Supplementary Material**

**Comparison of motion grading in 1,000 patients by first- and second-generation HR-pQCT: A propensity score matched cohort study**

Mikolaj Bartosik, Alexander Simon, André Strahl, Ralf Oheim, Michael Amling, Felix N. Schmidt

**Supplementary Table 1 Overview of the entire study cohort.**

|  |  |  | **XCT 1** (n = 500) | | | |  | **XCT 2** (n = 500) | | | |  |  |
| --- | --- | --- | --- | --- | --- | --- | --- | --- | --- | --- | --- | --- | --- |
| Parameter | | | Mean | SD | Min | Max |  | Mean | SD | Min | Max | *p* | r \| φ |
| **Demographics** | | |  |  |  |  |  |  |  |  |  |  |  |
|  | Female (%) | | 78.8 |  |  |  |  | 70.4 |  |  |  | **0.002** | 0.10 |
|  | Age (years) | | 66.9 | 12.4 | 20 | 89 |  | 60.4 | 15.5 | 20 | 87 | **< 0.001** | 0.22 |
|  | Weight (kg) | | 66.8 | 14.4 | 38.2 | 146.0 |  | 70.6 | 16.1 | 41.1 | 155.3 | **< 0.001** | 0.12 |
|  | Height (m) | | 1.67 | 0.09 | 1.46 | 1.95 |  | 1.69 | 0.09 | 1.38 | 1.98 | **< 0.001** | 0.13 |
|  | BMI (kg/m^2^) | | 23.9 | 4.4 | 15.4 | 50.5 |  | 24.6 | 4.7 | 15.7 | 54.4 | **0.020** | 0.07 |
| **DXA** | | |  |  |  |  |  |  |  |  |  |  |  |
|  | Spinal T-score | | -1.7 | 1.5 | -5.1 | 4.8 |  | -1.0 | 1.7 | -4.3 | 4.9 | **< 0.001** | 0.19 |
|  | Spinal Z-score | | -0.3 | 1.6 | -4.0 | 6.4 |  | -0.1 | 1.7 | -3.8 | 6.4 | 0.086 | 0.05 |
|  | Femoral T-score | | -2.1 | 0.9 | -4.2 | 3.3 |  | -1.7 | 1.1 | -4.8 | 2.3 | **< 0.001** | 0.19 |
|  | Femoral Z-score | | -0.8 | 1.0 | -3.3 | 5.1 |  | -0.7 | 1.0 | -4.6 | 3.8 | 0.394 | 0.03 |
|  | Lowest T-score | | -2.4 | 1.0 | -5.1 | 3.3 |  | -2.0 | 1.2 | -4.8 | 2.2 | **< 0.001** | 0.19 |
|  | Lowest Z-score | | -1.1 | 1.0 | -4.0 | 5.1 |  | -1.0 | 1.1 | -4.6 | 3.8 | 0.496 | 0.02 |
|  | Normal BMD | | 38 of 500 (7.6%) | | | |  | 87 of 500 (17.4%) | | | | **< 0.001** | 0.15 |
|  | Osteopenia (< -1.0) | | 205 of 500 (41.0%) | | | |  | 231 of 500 (46.2%) | | | | 0.097 | 0.05 |
|  | Osteoporosis (≤ -2.5) | | 257 of 500 (51.4%) | | | |  | 182 of 500 (36.4%) | | | | **< 0.001** | 0.15 |
| **Mechanography** | | |  |  |  |  |  |  |  |  |  |  |  |
|  | Grip strength (kg) | | 25.3 | 8.9 | 7.6 | 63.5 |  | 27.2 | 9.5 | 7.9 | 64.9 | **< 0.001** | 0.11 |
|  | CRT maximum force (kN) | | 0.87 | 0.19 | 0.44 | 1.93 |  | 0.92 | 0.21 | 0.47 | 1.97 | **0.003** | 0.09 |
|  | CRT time per repetition (s) | | 1.96 | 0.81 | 0.76 | 8.30 |  | 2.09 | 0.97 | 0.82 | 9.42 | **0.025** | 0.07 |
|  | Romberg path length EO (mm) | | 145.1 | 56.4 | 51.7 | 420.2 |  | 137.7 | 58.0 | 52.9 | 422.1 | **0.004** | 0.09 |
|  | Romberg path length EC (mm) | | 229.4 | 118.4 | 68.1 | 981.9 |  | 219.6 | 109.2 | 61.2 | 853.7 | 0.162 | 0.04 |
|  | Normal muscle performance | | 406 of 500 (81.2%) | | | |  | 409 of 500 (81.8%) | | | | 0.807 | 0.01 |
|  | Sarcopenia | | 94 of 500 (18.8%) | | | |  | 91 of 500 (18.2%) | | | |  |  |

Normal bone mineral density (BMD), osteopenia, and osteoporosis were categorized based on the T-score. Sarcopenia was classified according to the sarcopenia EWGSOP2 consensus scores (*A.J. ­­­Cruz-Jentoft, et al., Sarcopenia: revised European consensus on definition and diagnosis. Age Ageing, 2019. 48(1):16-31*).

SD: standard deviation; BMI: body mass index; DXA: dual-energy X-ray absorptiometry; CRT: chair-rising test; EO: eyes open; EC: eyes closed.

Numbers in bold indicate statistical significance (*p* < 0.05) and effect sizes were reported as r or φ.

**Supplementary Table 2 Bone microarchitecture of the distal radius and fibula assessed by HR-pQCT in patients with adequate motion grading (Grades 1-3).**

|  |  | **XCT1** | | |  | **XCT2** | | |  | **XCT1** | | |  | **XCT2** | | |
| --- | --- | --- | --- | --- | --- | --- | --- | --- | --- | --- | --- | --- | --- | --- | --- | --- |
|  |  | **Radius** (n = 259) | | |  | **Radius** (n = 338) | | |  | **Tibia** (n = 379) | | |  | **Tibia** (n = 376) | | |
| Parameter | | Mean | SD | % median |  | Mean | SD | % median |  | Mean | SD | % median |  | Mean | SD | % median |
| **HR-pQCT** | |  |  |  |  |  |  |  |  |  |  |  |  |  |  |  |
|  | Tt.BMD (mg HA/cm^3^) | 260.7 | 60.5 | 83.9 |  | 243.2 | 62.8 | 77.7 |  | 231.7 | 51.7 | 82.1 |  | 225.8 | 55.7 | 76.7 |
|  | Tt.Ar (mm^2^) | 303.3 | 74.7 | 103.0 |  | 296.8 | 69.1 | 108.3 |  | 772.8 | 140.5 | 107.0 |  | 768.3 | 155.4 | 110.4 |
|  | Tb.BMD (mg HA/cm^3^) | 127.9 | 40.7 | 78.8 |  | 115.5 | 42.4 | 77.3 |  | 141.2 | 36.3 | 82.3 |  | 135.9 | 40.8 | 82.5 |
|  | BV/TV | 0.107 | 0.034 | - |  | 0.170 | 0.056 | 83.9 |  | 0.118 | 0.030 | - |  | 0.209 | 0.052 | 87.6 |
|  | Tb.N (mm^-1^) | 1.67 | 0.42 | 84.7 |  | 1.135 | 0.303 | 83.6 |  | 1.64 | 0.39 | 89.6 |  | 1.145 | 0.259 | 88.7 |
|  | Tb.Th (mm) | 0.064 | 0.013 | 85.7 |  | 0.227 | 0.019 | 99.6 |  | 0.073 | 0.014 | 93.8 |  | 0.256 | 0.024 | 99.2 |
|  | Tb.Sp (mm) | 0.598 | 0.293 | 135.1 |  | 0.967 | 0.509 | 136.6 |  | 0.584 | 0.224 | 124.2 |  | 0.942 | 0.484 | 125.9 |
|  | Tb.Ar (mm^2^) | 242.8 | 68.8 | 105.1 |  | 247.4 | 64.3 | 114.5 |  | 661.6 | 138.2 | 110.1 |  | 669.8 | 153.1 | 117.1 |
|  | Ct.BMD (mg HA/cm^3^) | 762.4 | 74.2 | 82.1 |  | 827.7 | 83.7 | 92.6 |  | 743.0 | 72.8 | 85.5 |  | 787.1 | 81.5 | 91.3 |
|  | Ct.Th (mm) | 0.61 | 0.19 | 66.8 |  | 0.869 | 0.198 | 83.2 |  | 0.81 | 0.27 | 67.4 |  | 1.167 | 0.284 | 79.0 |
|  | Ct.Po | - | - | - |  | 0.008 | 0.006 | 125.4 |  | - | - | - |  | 0.033 | 0.015 | 136.0 |
|  | Ct.Pm (mm) | 74.5 | 9.8 | - |  | 73.2 | 9.0 | - |  | 109.7 | 10.3 | - |  | 108.4 | 11.1 | - |
|  | Ct.Ar (mm^2^) | 45.1 | 15.2 | 78.9 |  | 53.3 | 13.0 | 87.2 |  | 88.4 | 30.2 | 79.0 |  | 104.1 | 26.3 | 80.3 |

HR-pQCT results of the distal radius and tibia were compared to the median of device-, age- and sex-specific reference values (XCT1: *L.A. Burt, et al., Sex- and Site-Specific Normative Data Curves for HR-pQCT. J Bone Miner Res, 2016. 31(11):2041-2047*, XCT2: *D.E. Whittier, et al., Sex- and Site-Specific Reference Data for Bone Microarchitecture in Adults Measured Using Second-Generation HR-pQCT. J Bone Miner Res, 2020. 35(11):2151-2158*).

HR-pQCT: high-resolution peripheral quantitative computed tomography; Tt.BMD: total BMD; HA: hydroxyapatite; Tt.Ar: total area; Tb.BMD: trabecular BMD; BV/TV: bone volume to tissue volume; Tb.N: trabecular number; Tb.Th: trabecular thickness; Tb.Sp: trabecular separation; Tb.Ar: trabecular area; Ct.BMD: cortical BMD; Ct.Th: cortical thickness; Ct.Po: cortical porosity (pore volume to total volume ratio); Ct.Pm: cortical perimeter; Ct.Ar: cortical area.
